# Targeting the immuno-inflammatory-microbial network: a key strategy for sepsis treatment

**DOI:** 10.3389/fimmu.2025.1575516

**Published:** 2025-04-14

**Authors:** Yue Xu, Jiaxin Wang, Rui Yuan, Zhu Qin, Kunlan Long, Peiyang Gao

**Affiliations:** Hospital of Chengdu University of Traditional Chinese Medicine, Chengdu, China

**Keywords:** cytokine storm, immune balance, probiotics, treatment outcome, traditional Chinese medicine (TCM)

## Abstract

Sepsis is a life-threatening condition caused by a dysregulated host response to infection, remaining a major global health challenge despite clinical advances. Therapeutic challenges arise from antibiotic misuse, incomplete understanding of its complex pathophysiology, and the unresolved interplay of immune dysregulation and microbiota disruption. Investigating microbial homeostasis in the shift from cytokine storm to immunosuppression may elucidate the interplay between microbial metabolites, immune dysfunction, and organ injury, providing a foundation for targeted therapies and drug development. Traditional Chinese Medicine (TCM) has demonstrated significant advantages in mitigating sepsis-associated cytokine storms and modulating gut microbiota homeostasis, offering a promising strategy for developing highly effective and less toxic targeted monomeric compounds. Elucidating the interactions within the immune-inflammation-microbiota network in sepsis paves the way for biomarker-driven personalized therapeutic approaches.

## 1 Introduction

Sepsis is a systemic inflammatory response syndrome (SIRS) triggered by infection, fundamentally characterized by an uncontrolled immune response to pathogens, resulting in immune dysregulation, multiple organ dysfunction, and potentially life-threatening conditions (1). According to the 2016 Sepsis-3.0 consensus definition (2, 3), sepsis is defined as life-threatening organ dysfunction caused by a dysregulated host response to infection (4), typically quantified by an increase in the Sequential Organ Failure Assessment (SOFA) score of ≥2 points (5). Sepsis imposes a substantial global health burden, affecting approximately 48.9 million individuals and accounting for 20% of all global deaths in 2017 (6).

The mortality rate of non-shock sepsis ranges from 25% to 30%, while septic shock, characterized by profound circulatory and metabolic abnormalities, has a mortality rate of approximately 42.3% (7–9). The prevention, management, and prognosis of sepsis are influenced by regional economic status and healthcare resources (10). Patients in high-income countries (HICs) generally have better outcomes, whereas those in low- and middle-income countries (LMICs) face higher rates of misdiagnosis and adverse outcomes due to resource limitations and reduced efficiency in sepsis recognition and management (6). Moreover, non-infectious inflammatory conditions, such as trauma and pancreatitis, can mimic sepsis symptoms, leading to misdiagnosis rates of 15–40% (11, 12). Therefore, the development of more effective treatment strategies and the improvement of sepsis diagnostic accuracy are critical approaches to reducing sepsis-related mortality.

The key to improving diagnostic accuracy lies in elucidating the specific pathological mechanisms and etiologies of sepsis. A multidimensional integrated therapeutic strategy based on the immune-inflammatory-microbial regulatory network, including targeting cytokine storms, modulating immune cell dysfunction, and restoring microbial homeostasis, serves as the critical scientific foundation for mitigating sepsis-associated tissue damage and improving patient outcomes.

During the progression of sepsis, pathogenic microorganisms interact with host pattern recognition receptors (PRRs) (13), through their pathogen-associated molecular patterns (PAMPs) (14). activating signaling pathways such as nuclear factor-kappa B (NF-κB), mitogen-activated protein kinase (MAPK), and JAK-STAT, thereby triggering acute inflammatory responses (15), Immune cells, including macrophages, neutrophils, and dendritic cells, secrete cytokines and chemokines such as TNF-α, IL-1β, and IL-6, which amplify the immune response (16).

As sepsis progresses, the excessive activation of the immune-inflammatory response not only exacerbates the cytokine storm but also triggers complex feedback mechanisms within the immune system (17), with immunosuppression emerging as the most common pathological process in the later stages of sepsis (18). Elucidating the key mechanisms underlying immunosuppression is critical for the development of targeted therapies and the implementation of precision medicine. During the pathophysiological process of sepsis, the interaction between pathogenic microorganisms and host immune cells forms a dynamic and tightly interconnected microbiota-immune network (19). Microbiota dysbiosis stimulates inflammatory responses through the Toll-like receptor 4 (TLR4)/myeloid differentiation primary response 88 (MyD88) signaling pathway (20) and impairs intestinal barrier function via the phosphoinositide 3-kinase (PI3K)/Akt pathway (21), facilitating the translocation of pathogenic bacteria into the bloodstream and exacerbating systemic inflammation (22, 23). The dynamic interplay between inflammation and immunosuppression plays a pivotal role in the progression of sepsis (24), not only determining the pathological trajectory but also directly influencing clinical outcomes. Therefore, a comprehensive understanding of the immune-inflammatory-microbiota network (25), particularly the interactions among signaling pathways and regulatory factors, provides a critical foundation for the development of effective therapeutic strategies.

This review summarizes the dysregulation of the immune-inflammatory-microbiota network and its key mechanisms in the progression of sepsis. It analyzes the interactions among immune responses, inflammatory reactions, and microbiota dysbiosis, and explores how these mechanisms offer new therapeutic targets and potential strategies for sepsis treatment. By delving into the underlying mechanisms of this network, the review provides new perspectives for precision therapeutic approaches in sepsis, aiming to advance treatment strategies and improve clinical outcomes.

## 2 Review methodology

**Figure 1:**
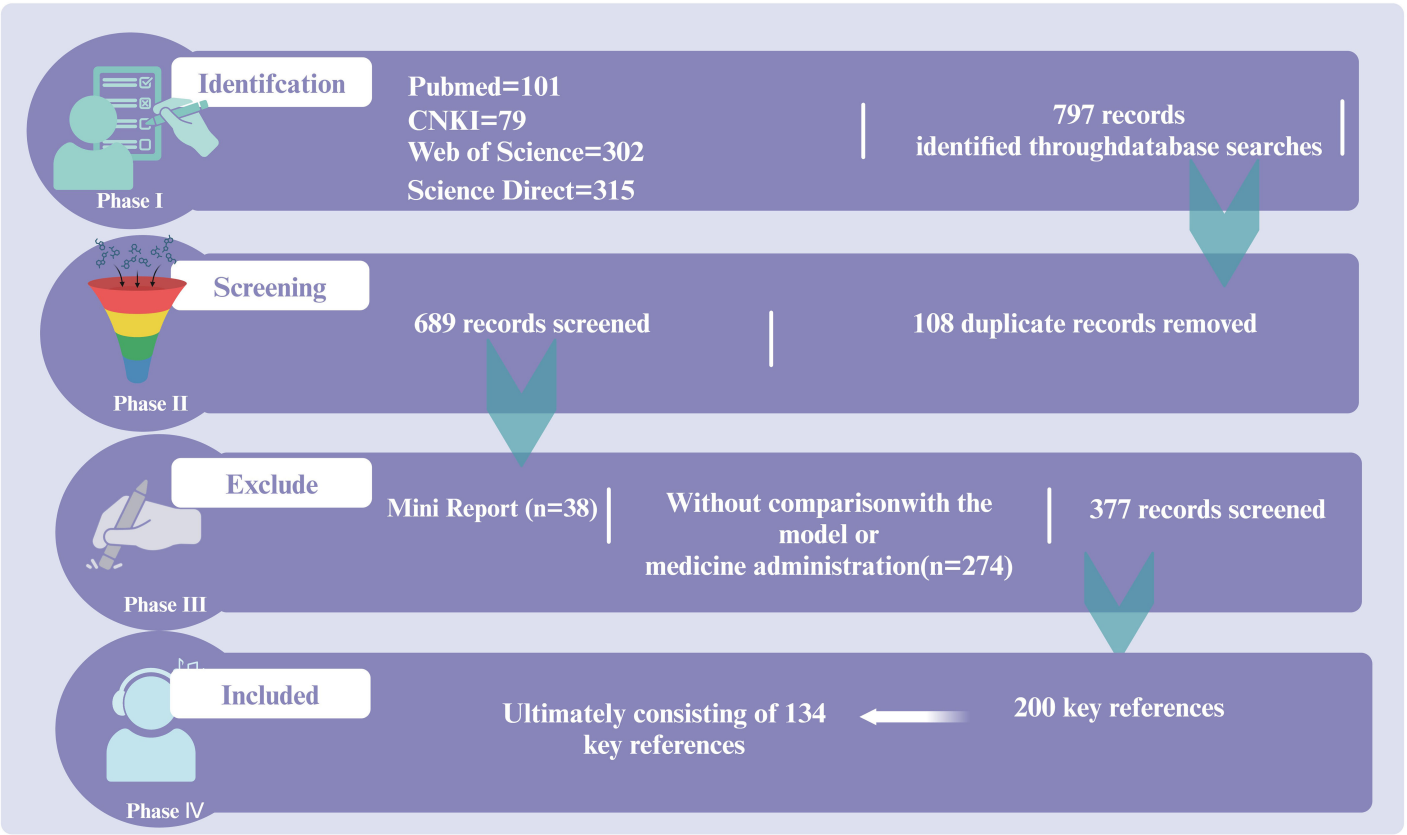
The literature search and screening flowchart.

A review was conducted on the regulatory role of the immune-inflammation-microbiota network and its mechanisms in sepsis treatment based on the guidelines of the Society of Critical Care Medicine (SCCM) and the European Society of Intensive Care Medicine (ESICM). Data were collected from research articles published before March 2020 in Web of Science, PubMed, ScienceDirect, and CNKI. This information was utilized to summarize the critical roles of immunity, inflammation, and microbiota in sepsis progression, as well as the activators and inhibitors of key targets, with a particular emphasis on the regulatory effects of traditional Chinese medicine monomers on sepsis. The inclusion criteria were as follows: (a) clinical studies; (b) reviews discussing sepsis-related mechanisms, including inflammation, immunity, and microbiota. The exclusion criteria included: (a) mini-reports; (b) studies without comparisons with models; (c) duplicate articles. The review focused on keywords present in the title, abstract, or full text to ensure research reliability. A total of 157 articles were included for review (**Figure 1**).

## 3 Pathophysiological mechanisms of sepsis

### 3.1 Early cytokine storm and immune homeostasis imbalance in sepsis

Early biomarkers of sepsis include C-reactive protein (CRP) and procalcitonin (PCT) (26), which help distinguish bacterial infections from sterile inflammation. Soluble triggering receptor expressed on myeloid cells-1 (sTREM-1) serves as an indicator of innate immune activation and is significantly elevated in bacterial sepsis (27). Additionally, CD64 expression levels reflect neutrophil activation (28), providing potential value for early sepsis diagnosis. The early stage of systemic inflammatory response syndrome (SIRS) is marked by a robust cytokine storm, which can subsequently evolve into compensatory anti-inflammatory response syndrome (CARS) or persistent inflammation (29), immunosuppression, and catabolism syndrome (PICS) (30). Throughout this pathological progression, maintaining the dynamic equilibrium of the immune regulatory network is critical. Achieving immune homeostasis necessitates the precise spatiotemporal modulation of pro-inflammatory mediators (IL-1β, TNF-α, and IL-6) alongside anti-inflammatory mediators (IL-10 and TGF-β) (18).

The biological foundation of this therapeutic approach relies on an in-depth understanding of the functional interplay within the network of innate immune cells, including macrophages (31), neutrophils (32), and dendritic cells (33). In sepsis, microbiota dysbiosis exacerbates the aberrant activation of these cells through the gut-immune axis (34). The disruption of this balance impairs the normal functioning of the immune response, leading to an immunosuppressive state that further exacerbates immune dysfunction in sepsis.

### 3.2 Mechanisms of macrophage pro-inflammatory to immune suppressive transition in sepsis

Macrophages exhibit a dynamic dual regulatory role in the pathophysiology of sepsis. During the early stages of infection, macrophages recognize PAMPs (35), such as LPS from Gram-negative bacteria, through Toll-like receptors (TLRs), thereby initiating the immune response (36).

This process involves the activation of both MyD88-dependent and TRIF-dependent signaling pathways (37). Upon immune activation, the nuclear translocation of NF-κB and interferon regulatory factors (IRFs) occurs (38), promoting the transcription and secretion of pro-inflammatory cytokines such as TNF-α, IL-1β, and IL-6. Additionally, chemokines mediate the recruitment of immune cells, including neutrophils and monocytes, to the site of infection, thereby initiating a local inflammatory response to eliminate the pathogens (31). As sepsis progresses, macrophages transition from classically activated M1 macrophages to alternatively activated M2 macrophages (16). This shift is accompanied by the negative regulation of NF-κB and MAPK signaling pathways (39), resulting in a decrease in pro-inflammatory cytokine production and an upregulation of immunosuppressive mediators, such as interleukin-10 (IL-10) and TGF-β. Concurrently, immune checkpoint molecules (ICMs) such as programmed cell death protein-1 (PD-1) (40) and T-cell immunoglobulin and mucin domain-containing molecule-3 (TIM-3) are significantly upregulated on the surface of macrophages (41). These molecules transmit inhibitory signals that further impair the antigen-presenting capacity of macrophages, contributing to the suppression of the immune response.

The establishment of this immunotolerant state is closely linked to epigenetic modifications, such as histone deacetylation, and metabolic reprogramming, such as the shift toward aerobic glycolysis. These processes ultimately lead to immunoparalysis (42), which increases the risk of secondary infections and promotes the onset and progression of multiple organ dysfunction syndrome (MODS) (43).

### 3.3 Dual role of neutrophils in immune defense and tissue injury in sepsis

Neutrophils play a dual role in the immune pathological process of sepsis, acting both as key effector cells of innate immune defense and as important participants in inflammation-mediated tissue damage (32). As the first immune effector cells recruited to the site of infection, neutrophils recognize PAMPs via TLRs and other PRRs, initiating intracellular signaling cascades (44). By activating the NF-κB and MAPK pathways, they release pro-inflammatory cytokines TNF-α, IL-1β, chemokine CXCL8, and antimicrobial peptides such as cathelicidins (45), thereby forming a local inflammatory microenvironment to eliminate pathogens.

During the acute phase of sepsis, neutrophil functions such as phagocytosis, degranulation, and respiratory burst activity are enhanced, processes that play a critical role in pathogen clearance. As the disease progresses, neutrophil dysfunction occurs, leading to an overactivation of the immune response accompanied by persistent inflammation (46). This abnormal state results in the release of large amounts of reactive oxygen species (ROS) and proteolytic enzymes (such as elastase and matrix metalloproteinases, MMPs) (47), which directly damage endothelial cells through oxidative stress and proteolysis, compromising the integrity of the endothelial barrier. Concurrently, microvascular permeability increases, and extracellular matrix (ECM) components are degraded (48), exacerbating the SIRS and multiple organ dysfunction (MOD). Neutrophils participate in the pathological progression of sepsis through the formation of neutrophil extracellular traps (NETs) (49). The formation of NETs relies on histone citrullination and the release of granzyme, with their structure composed of chromatin DNA, histones, and granule proteins. Although NETs exert antibacterial effects by capturing pathogens, their excessive release can lead to microvascular thrombosis and tissue ischemia-reperfusion injury (50). Additionally, NETs activate the complement system and TLR signaling pathways, promoting the cascade release of pro-inflammatory cytokines. This forms a positive feedback loop, further exacerbating immune imbalance and tissue damage. NETs-associated molecules, such as cfDNA and MPO-DNA complexes, can also serve as damage-associated molecular patterns (DAMPs), which exacerbate the inflammatory response by activating pathways like the NLRP3 inflammasome (51). Therefore, neutrophil dysregulation and abnormal activation play a key role in sepsis and are important driving factors of sepsis-associated multiple organ failure (MOF). Targeting the regulation of neutrophil activation, NET formation, and the inflammation-mediated damage they induce may offer new therapeutic intervention targets for sepsis.

### 3.4 Dual role of dendritic cells in immune activation and tolerance in sepsis

Dendritic cells (DCs), as key antigen-presenting cells (APCs), play a crucial role in the pathological progression of sepsis by recognizing PAMPs via PRRs (52). This recognition triggers antigen processing and presentation, thereby activating specific immune responses. In the early stages of sepsis, dendritic cells (DCs) promote Th1-type immune polarization through the expression of TLRs and C-type lectin receptors (CLRs) on their surface (53). They secrete pro-inflammatory cytokines, such as IL-12 and IL-6, enhancing the host’s response to pathogens.

However, as sepsis progresses to its late stages, dendritic cells (DCs) undergo a functional phenotypic shift from an immune activation state to an immune tolerance state. This process is closely related to the negative feedback regulation induced by the excessive activation of TLR signaling pathways. Additionally, DCs are regulated by immune-suppressive mediators secreted by myeloid-derived suppressor cells (MDSCs) (54) and regulatory T cells (Tregs), such as transforming growth factor-β (TGF-β) and IL-10 (55). Refer to **Table 1** for information about immune factors that play a key role in sepsis. The dysfunction of DCs not only weakens pathogen clearance efficiency but also promotes the expression of immune checkpoint molecules (such as PD-1 and CTLA-4) (56), inhibiting T cell activation and leading to acquired immune deficiency.

**Table 1:** Key immunological mediators in sepsis.

Name	Type	Function	Target	Mechanism	Drug	Dosage	References
IFN-γ	Immune factor	Enhances macrophage phagocytosis and bactericidal activity	mHLA-DR, TNF-α	JAK-STAT pathway	Interferon gamma-1b	50-100 mg/kg	(58)
GM-CSF	Immune factor	Boosts neutrophil, monocyte, and macrophage activity in sepsis	mHLA-DR, CD11b	PI3K/Akt pathway	Leukine (GM-CSF)	125 µg/m^2^	(59)
IL-7	Immune factor	Promotes T-cell proliferation and inhibits apoptosis	CD4, CD8 T-cells	JAK-STAT pathway	Kymriah (IL-7)	1-2 μg/ml	(60)
Thymosin α1 (Tα1)	Immunomodulator	Activates immune cells (e.g., DC, NK cells, macrophages) and increases T-cells	CD4, CD8 T-cells, PD-1, TIM-3	TLR, NF-κB pathway	Thymalfasin (Tα1)	1.6 mg/Week	(61)
Mesenchymal Stem Cells (MSCs)	Cell Therapy	Improves bacterial clearance, modulates immune response, reduces apoptosis	LL-37, NLRP3, TNF-α, IL-6, M2 Macrophages, Tregs	TLR, NF-κB, PI3K/Akt pathways	MSC Therapy	As per treatment plan	(62)
Immune Checkpoint Inhibitors	Immunomodulator	Restores immune cell function and enhances infection resistance	TIM-3, PD-1, PD-L1, BTLA	PD-1/PD-L1 pathway	Durvalumab, PD-L1 inhibitor	As per treatment plan	(63)
IL-10	Immune factor	Suppresses inflammation and modulates immune tolerance	TNF-α, IL-6, NF-κB	JAK-STAT pathway	N/A	N/A	(63)
TGF-β	Immune factor	Inhibits immune cell activation and promotes immune tolerance	Smad2/3, NF-κB	TGF-β/Smad pathway	TGF-β mRNA	10 μM	(64)
IL-2	Immune factor	Enhances T-cell proliferation and immune response	CD8 T-cells, IL-2R	JAK-STAT pathway	Proleukin	9 × 10^6^ IU	(65)
IL-12	Immune factor	Promotes Th1 cell differentiation and cell-mediated immunity	NK cells, T-cells	JAK-STAT, PI3K/Akt	IL-12 mRNA	2 μg	(66)
IL-15	Immune factor	Enhances T-cell proliferation and NK cell activity	NK cells, CD8 T-cells	JAK-STAT, PI3K/Akt	Recombinant anti-IL-15 antibody	200 ug/kg	(67)
TNF-α	Immune factor	Activates immune response and enhances inflammation	NF-κB, MAPK pathway	NF-κB, MAPK	N/A	N/A	(68)
IL-1β	Immune factor	Initiates inflammatory response and activates immune system	NF-κB, MAPK pathway	NF-κB, MAPK	N/A	N/A	(69)

N/A, Not Applicable (indicating that the data is irrelevant or unavailable in this context).

**Figure 2:**
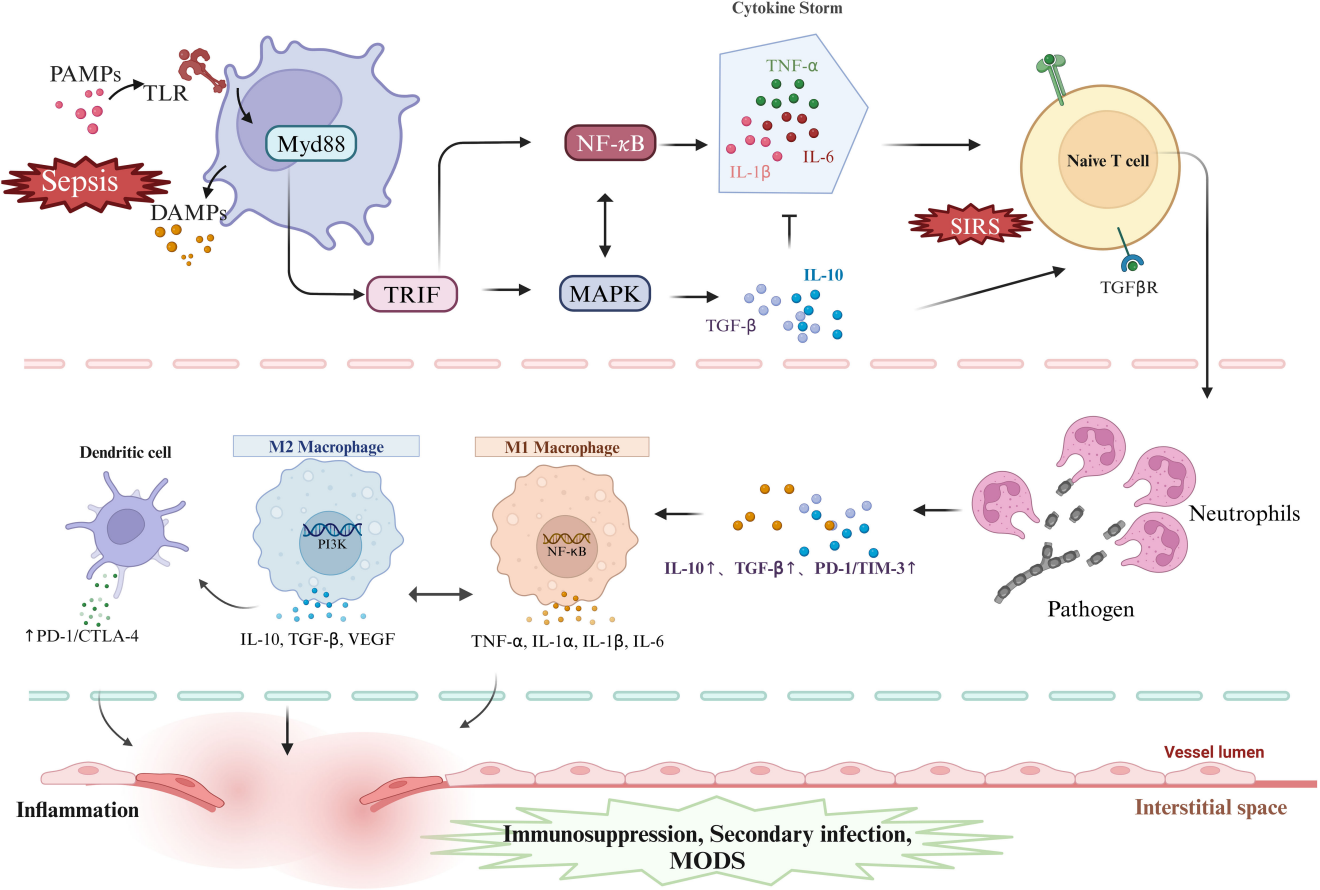
DAMPs and PAMPs activate PRRs, triggering MyD88- and TRIF-dependent pathways, which in turn activate NF-κB and MAPK signaling, driving the release of pro- and anti-inflammatory cytokines and promoting T cell activation. Neutrophils regulate inflammation by sensing cytokine shifts and enhancing anti-inflammatory responses. In sepsis, macrophages shift from M1 to M2 phenotypes, balancing inflammation and repair but risking immunosuppression and secondary infections. PAMPs, pathogen-associated molecular patterns; TLR, Toll-like receptors; Myd88, myeloid differentiation factor 88;TRIF,Toll-like receptor interleukin-1 receptor domain-containing adaptor inducing interferon-β; MAPK, mitogen-activated protein kinase; NF-κB, nuclear transcription factor-kB; TNF-α, tumor necrosis factor-alpha; IL-6,interleukin-6; IL-1α,interleukin-1α; IL-1β, interleukin-1β; IL-10, interleukin-10; TGF-β, transforming growth factor-β; TGF-βR, transforming growth factor-β receptor; SIRS, Systemic Inflammatory Response Syndrome; PD-1, programmed cell death protein 1; CTLA-4, Cytotoxic T Lymphocyte-Associated Antigen-4; TIM-3, T cell immunoglobulin and mucin domain-containing protein 3; MODS, Multiple Organ Dysfunction Syndrome; PI3K, Phosphatidylinositol 3-kinase. Created in https://BioRender.com.

Moreover, tolerant DCs further reinforce the immunosuppressive microenvironment by secreting immune-suppressive cytokines, such as IL-10 and TGF-β, and inducing the expansion of Tregs, thus forming a positive feedback loop (33). The persistence of this immune tolerance state promotes the chronic progression of sepsis, increasing the risk of secondary infections and accelerating the development of multiple organ dysfunction syndrome (MODS) (57). Therefore, DCs play a dual role in the immune regulatory network of sepsis, acting as a bridge between innate and adaptive immunity, while also serving as central regulators of the immunosuppressive state. Three cell types play critical roles in the progression of sepsis. Their specific mechanisms and interactions are illustrated in **Figure 2**.

## 4 Immune balance in sepsis

### 4.1 Immune imbalance and regulation via PAMPs and PRRs

The inflammatory response in sepsis is triggered by the interaction between PAMPs and host PRRs. PAMPs, such as bacterial LPS, viral single-stranded RNA, and fungal β-glucans, bind to PRRs (70), thereby activating the host immune response and initiating a systemic inflammatory reaction. TLR4, as a key receptor, mediates the effects of LPS by activating the MyD88- dependent signaling pathway (71). This activation subsequently triggers extracellular signal-regulated kinases (ERK1/2), p38, c-Jun N-terminal kinase (JNK), and NF-κB, leading to the synthesis and secretion of pro-inflammatory cytokines such as IL-1β, TNF-α, and IL-6 (72). These pro-inflammatory cytokines circulate to tissues and organs, activating immune cells and amplifying inflammation. Endogenous HMGB1 is released extracellularly during cell injury or death, activating TLRs and RAGE receptors and regulating NF-κB and MAPK pathways to trigger inflammation (73). HMGB1 plays a key role in inflammasome activation, autophagy, cell survival, coagulation, and immune regulation. Studies have shown that macrophage activation under LPS stimulation is closely associated with HMGB1 release, in which oxidative stress and calcium signaling play critical roles (74). Post-translational modifications of HMGB1 not only contribute to inflammasome activation but also regulate cell death pathways such as pyroptosis (75). These mechanisms interact synergistically, exacerbating inflammation and immune dysregulation in sepsis.

Different types of PAMPs and DAMPs activate multiple signaling pathways through PRRs, collectively driving the pathogenesis of sepsis and promoting the interplay between systemic inflammatory responses and immunosuppressive states. The key information regarding DAMPs, PAMPs, and immune regulatory factors is summarized in **Table 2**.

**Table 2:** Key information on different damps, pamps, and immune regulatory factors in sepsis.

Factor Name	Type	Roles	Source	Primary Actions	Agonists/Inhibitors	Action pathway	Mechanism of Action and Effect	Reference
LSECs (Liver sinusoidal endothelial cells)	immunomodulator	Leukocyte migration	Liver	Phagocytosis, antigen presentation	Agonists: TNF-α, IFN-γ	NF-κB, S1P	Modulates immune cell migration, tolerance	(76)
Macrophage Polarization	immunomodulator	Immune regulation	Bone, spleen	M1/M2 activation, cytokine release	Agonists: IFN-γ (M1), IL-4 (M2); Inhibitors: IL-10, TGF-β	M1/M2 polarization, NF-κB, MAPK	M1: inflammation; M2: tissue repair	(77)
KCs-Platelet Interaction	immunomodulator	Platelet aggregation,	Liver, blood	Immune cell recruitment	Agonists: PAF; Inhibitors: Aspirin	Platelet signaling, TLR4/MD-2, MAPK	Anti-inflammatory, thrombosis	(19)
Neutrophils	immunomodulator	Pathogen killing	Bone, blood	Phagocytosis, ROS release, cytokine secretion	Agonists: IL-8, GM-CSF; Inhibitors: Corticosteroids	NF-κB, ROS, NET formation	Neutrophil survival, pathogen clearance	(78)
NK Cells and NKT Cells	immunomodulator	Infected cell killing, immune regulation	Spleen, blood	Cytotoxicity, cytokine secretion	Agonists: IL-12, IL-15; Inhibitors: IL-10	NKG2D/NCR, NF-κB, mTOR	Cytotoxic killing, immune regulation	(79)
Hepatocytes	immunomodulator	Acute-phase protein production, detoxification	Liver	Complement protein synthesis, cytokine release	Agonists: IL-6, TNF-α	JAK/STAT, NF-κB	Protein synthesis, metabolic regulation	(80)
LPS	immunomodulator	Pathogen detection, inflammation	Gram-negative bacteria	TLR4 activation, cytokine production	Inhibitors: LPS receptor antagonists	TLR4, NF-κB, MAPK	LPS inhibitors modulate immune response	(81)
Vagal Nerve	immunomodulator	Inflammation modulation	Vagus nerve	Inflammation suppression, cytokine regulation	Agonists: VNS	Cholinergic anti-inflammatory, NF-κB suppression	Reduces systemic inflammation	(82)
HMGB1	DAMPs	Inflammation, immune activation	Stressed/dying cells	TLR4/MyD88 activation, systemic inflammation	Inhibitors: Anti-HMGB1 antibodies	TLR4/MyD88, NF-κB	HMGB1 inhibitors reduce inflammation	(83)
ATP	DAMPs	Inflammasome activation	Dying cells/tissues	P2X receptor activation, NF-κB, MAPK signaling	Inhibitors: P2X receptor antagonists	NF-κB, MAPK	ATP antagonists suppress inflammation	(84)
S100 Proteins	DAMPs	Cytokine release, leukocyte migration	Immune cells, damaged tissues	RAGE, TLR4 activation, NF-κB, MAPK	Inhibitors: Anti-S100 antibodies	RAGE, TLR4, NF-κB, MAPK	Targeting S100 modulates inflammation	(85, 86)
Mitochondrial DNA	PAMPs	Inflammation promotion	Damaged mitochondria	TLR9/MyD88 activation, NF-κB, inflammasome	Inhibitors: Mitochondrial-targeted antioxidants	TLR9/MyD88, NF-κB	Antioxidants reduce inflammation	(87, 88)
RNA from Pathogens	PAMPs	Innate immune activation	Pathogens	RIG-I, TLR3 activation, antiviral response	Inhibitors: Antiviral agents	RIG-I, TLR3, NF-κB, JAK/STAT	Inhibits viral replication	(89, 90)
Peptidoglycan	PAMPs	Immune activation	Bacteria	TLR2 activation, cytokine release	Inhibitors: TLR2 antagonists	TLR2, NF-κB	Targeting TLR2 reduces bacterial-induced inflammation	(91)
Fungal β-glucans	PAMPs	Immune cell activation	Fungi	Dectin-1 activation, NF-κB, MAPK	Inhibitors: Dectin-1 inhibitors	Dectin-1, NF-κB, MAPK	Dectin-1 inhibitors manage fungal infections	(92, 93)

### 4.2 Core mechanisms of immune imbalance and multi-organ failure in sepsis

Cytokine-induced cytokine storms are a key feature in the progression of sepsis. In the early stages of sepsis, the binding of PAMPs to PRRs leads to excessive activation of immune cells (94). A large accumulation of inflammatory factors such as IL-1β, TNF-α, IL-6, and IL-8 occurs in tissues, with the activation of signaling pathways like NF-κB, MAPK, and JAK/STAT, enhancing cellular immune responses (95). When the immune system is excessively activated, it leads to a systemic inflammatory response, triggering multiple organ dysfunction syndrome (MODS) and microcirculatory disturbances. IL-6, as a central cytokine in the early inflammatory response of sepsis, exerts its effects through the JAK/STAT3 signaling pathway. Upon binding to its receptor, IL-6 activates JAK kinases and promotes the phosphorylation of the STAT3 transcription factor, which subsequently induces the secretion of CRP and PCT (26). The dramatic increase in these acute-phase proteins can serve as biomarkers for the diagnosis of sepsis. Within the threshold of homeostatic balance, these key factors enhance the immune response to combat infection. However, when this balance is disrupted, the excessive release of inflammatory cytokines such as TNF-α, IL-1β, and IL-6 leads to endothelial cell damage, increased vascular permeability, leukocyte infiltration, and the formation of a negative feedback loop that exacerbates the systemic inflammatory response in sepsis.

Within the threshold of homeostatic balance, these key factors enhance the immune response to combat infection. However, when this balance is disrupted, the excessive release of inflammatory cytokines such as TNF-α, IL-1β, and IL-6 leads to endothelial cell damage (96), increased vascular permeability, leukocyte infiltration, and the formation of a negative feedback loop that exacerbates the systemic inflammatory response in sepsis. Therefore, the cytokine storm and immune dysregulation are crucial interactive components in the pathological process of sepsis. Excessive release of pro-inflammatory cytokines disrupts immune balance, while immune suppression in the later stages of sepsis leads to the loss of the body’s defense mechanisms. Maintaining the balance of cytokine levels and the immune system may be key to reducing sepsis mortality in the future.

### 4.3 Inflammation-driven immune suppression in sepsis

The dynamic balance between immune function and inflammation is crucial, with immune suppression being key to improving patient prognosis. Understanding the specific mechanisms of excessive immune activation and immune suppression is fundamental for future research and clinical diagnosis. Immune suppression primarily occurs in the later stages of sepsis, characterized by a significant upregulation of PD-1 and cytotoxic T lymphocyte antigen 4 (CTLA-4), which suppresses T cell activation and proliferation, weakening the intensity of the immune response (40). At this stage, tissues counteract inflammation by promoting the expression of IL-10 and transforming growth factor-beta (TGF-β) (64), reducing the immune system’s response to infection. IL-10, as a key factor, inhibits the NF-κB and Janus kinase/signal transducer and activator of transcription (JAK/STAT) pathways, playing a crucial role during the immune suppression phase. TGF-β reduces the intensity of the immune response by inhibiting T cell activation and regulating the Th1/Th2 balance (97).

In addition to cytokines, macrophage polarization plays a crucial role in the interplay between immune suppression and inflammation. During sepsis, macrophages transition from the M1 phenotype (pro-inflammatory) to the M2 phenotype (immunosuppressive) (16), with M2 macrophages subsequently secreting immune-regulatory factors such as IL-10 and TGF-β to suppress inflammation and promote immune tolerance. When this balance is disrupted, the activity of T cells, B cells, and natural killer (NK) cells significantly declines, leading to a compromised inflammatory sensing system and an increased risk of secondary infections (79). Current clinical interventions for sepsis primarily focus on anti-inflammatory and immune-regulatory restoration. Studies have shown that TNF-α-targeting monoclonal antibodies such as Infliximab and Adalimumab can significantly reduce the excessive activation of the inflammatory response (98). However, these drugs often have immunosuppressive side effects, potentially increasing the risk of infections.

Monoclonal antibodies targeting PD-1 and CTLA-4 can reduce the risk of excessive immune activation in the early stages of immune suppression. While these strategies offer new treatment possibilities, their use in sepsis remains limited (99). Understanding the mechanisms of immune suppression-inflammation interplay is crucial for future research and clinical applications. Balancing the inhibition of excessive inflammation with the restoration of immune defense will be a key challenge in sepsis treatment.

## 5 Regulatory roles of the microbial network in sepsis

### 5.1 The critical role of gut microbiota in sepsis

In the immune dysregulation process of sepsis, the gut microbiota plays a key regulatory role in immune responses through its metabolic products, particularly short-chain fatty acids (SCFAs). SCFAs (100), including acetate, propionate, and butyrate, are produced by gut bacterial fermentation and play a crucial role in regulating immune cell functions, maintaining immune tolerance, and controlling systemic inflammation. SCFAs activate immune cells by binding to G-protein-coupled receptors GPR41 and GPR43. Studies have shown that acetate can promote the differentiation of T regulatory cells (Treg) through the GPR43 receptor, enhancing immune tolerance and suppressing pro-inflammatory responses by reducing the number of Th17 cells (101). In the context of sepsis, dysbiosis of the gut microbiota leads to further disruption of inflammation and immune responses, accompanied by a decrease in beneficial bacteria and excessive proliferation of pathogens, which in turn suppresses SCFA production and reduces the body’s ability to combat cachexia (101).

The primary mechanism of intestinal barrier damage involves the binding of LPS to the TLR4 receptor on cell surfaces during sepsis, which activates the downstream adaptor molecule MyD88 (81). This adaptor molecule recruits IRAK and TBK1, leading to the activation of the IκB kinase (IKK) complex. IKK promotes the phosphorylation and subsequent degradation of IκB, releasing NF-κB (38), which enhances the transcription of myosin light-chain kinase (MLCK). Increased phosphorylation of myosin light chain (MLC) promotes the contraction of actin-myosin filaments in the intestine, exacerbating intestinal permeability (102). This allows more bacteria, endotoxins, and PAMPs to infiltrate tissues, further triggering systemic inflammatory responses. Various cells and microorganisms are essential for intestinal function and health.

### 5.2 Restoring microbial homeostasis to regulate inflammation in sepsis

In sepsis, the gut microbiota plays a dual role: under homeostatic conditions, the microbiota maintains intestinal epithelial barrier integrity and regulates local immune homeostasis through competitive colonization, secretion of antimicrobial peptides (AMPs), and the production of metabolic products such as short-chain fatty acids (SCFAs) (103). During sepsis treatment, the overuse of antibiotics, nutritional support, and other clinical interventions often lead to dysbiosis. This promotes the overgrowth of pathogenic bacteria, such as *Enterococcus* spp, *Klebsiella pneumoniae*, and *Escherichia coli*, exacerbating systemic inflammatory responses and immune dysregulation (104).

**Figure 3:**
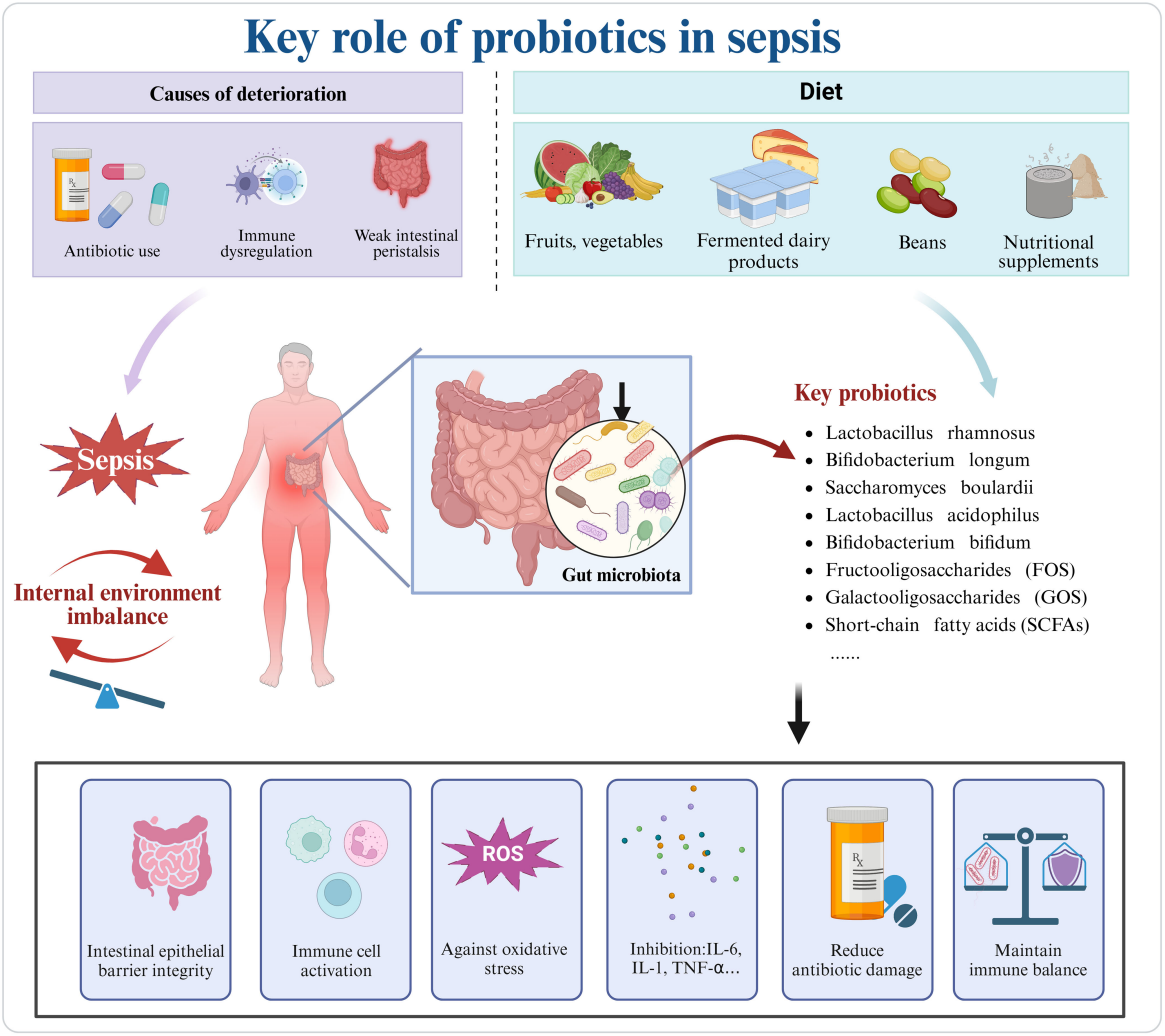
In sepsis, the misuse of antibiotics, immune dysregulation, and gastrointestinal motility issues exacerbate disease progression. Key probiotics, commonly supplemented through diet, can be obtained from vegetables, fruits, dairy products, legumes, and supplements. Probiotics can mitigate sepsis-induced damage by reconstructing the intestinal barrier, regulating immune cells, and combating oxidative stress. Created in https://BioRender.com.

Current therapeutic strategies for microbial disruption caused by sepsis include fecal microbiota transplantation (FMT), as well as supplementation with probiotics and prebiotics, aiming to restore intestinal microbial homeostasis (105, **Figure 3**). Studies have indicated that FMT can restore the relative abundance of *Firmicutes* and *Bacteroidetes* phyla in some septic patients, increase SCFA levels, and subsequently reduce serum levels of inflammatory markers, such as C-reactive protein and procalcitonin, as well as pro-inflammatory cytokines (105). The development of novel drug targets for microbiome-immune network regulation is currently a major research focus. Studies have revealed that PAMPs (such as LPS, bacterial RNA, and fungal β-glucan) and DAMPs (14), including HMGB1, ATP, and uric acid, regulate inflammation and immune cells through PRRs like TLR4, receptor for advanced glycation end products (RAGE), and TLR9. These interactions modulate key signaling pathways such as NF-κB (49). Gut homeostasis is influenced by *Methanobrevibacter smithii* (106), whose overgrowth during sepsis disrupts microbial balance and promotes bacterial translocation, ultimately compromising intestinal health. *Desulfovibrio* spp produces hydrogen sulfide (H_2_S) (107), which impairs the intestinal barrier and induces oxidative stress and inflammation in the septic environment. In summary, treatment strategies based on the immune-inflammation-microbiome network regulation, aimed at restoring a healthy microbial ecosystem and targeting key signaling pathways, are among the promising approaches to improve sepsis treatment efficacy. We have compiled key information on probiotics used in sepsis therapy, as shown in **Table 3**. Further mechanistic research and large-scale clinical trials will provide more evidence in this area and promote the clinical application of novel immunoregulatory therapies.

**Table 3:** Gut microecology in sepsis: probiotics, prebiotics, and other microorganisms.

Name	Main Action	Main Source	Dosage	References
*Lacticaseibacillus rhamnosus*	Strengthens barrier, modulates immunity, reduces inflammation.	Fermented dairy products (yogurt, cheese), supplements	1-10 billion CFU/day	(108)
*Bifidobacterium longum*	Reduces inflammation, strengthens mucosa, supports immunity.	Fermented dairy products, supplements	1-5 billion CFU/day	(109)
*Saccharomyces cerevisiae* var. *boulardii*	Regulates GALT, reduces permeability, inhibits pathogens.	Yeast, supplements	250-500 mg/day	(110)
*Lactobacillus acidophilus*	Strengthens gut barrier, modulates immunity.	Fermented dairy products, supplements	1-5 billion CFU/day	(111)
*Bifidobacterium bifidum*	Balances microbiome, boosts immunity, strengthens epithelium.	Fermented dairy products, supplements	1-10 billion CFU/day	(112)
Fructooligosaccharides (FOS)	Boosts good bacteria, increases SCFAs, supports gut health.	Fruits, vegetables	Scale add	(113)
Galactooligosaccharides (GOS)	Boosts gut bacteria, increases SCFAs, strengthens barrier.	Legumes, beans, dairy products	5-7 g/day	(114)
*Lactiplantibacillus plantarum*	Modulates immunity, reduces inflammation,	Fermented foods (sauerkraut, pickles), supplements	1-10 billion CFU/day	(115)
*Methanobrevibacter smithii*	Promote fiber fermentation, regulate gut microbiota balance	Human gut, especially colon	No fixed dosage	(106)
*Desulfovibrio* spp	Involved in sulfate metabolism, potentially affect gut barrier function and immune regulation	Gut, anaerobic environments	No fixed dosage	(107)
*Rotavirus* spp	Induces gut immune response, may promote mucosal immunity and microbiota stability	Human gut, contaminated food	No fixed dosage	(116)

## 6 TCM in gut microbiota and inflammation regulation in sepsis

Antibiotic therapy is a primary treatment for sepsis, but it has the side effect of disrupting the gut microbiota, potentially exacerbating the disease. In contrast, TCM has advantages in modulating microbial balance while addressing inflammation and immune responses. Xuebijing injection (XBJ), a Chinese herbal remedy, has been clinically approved for sepsis treatment. It primarily works by inhibiting NF-κB and GSK-3β signaling pathways (117), reducing pro-inflammatory cytokine release, and regulating the differentiation of Treg/Th17 cells in the treatment of sepsis.

Research has indicated that traditional Chinese medicine formulas, such as Dachengqi Decoction and Huanglian Jiedu Decoction (118), can improve intestinal barrier function and systemic immune response by repairing capillary permeability and regulating the TLR4/NF-κB and Akt/HO-1 signaling pathways. The combination of Huanglian can inhibit the inflammatory cascade induced by sepsis (119).

The use of individual herbs is also common, with species like Cordyceps sinensis, Rhizoma Coptidis (120), and Borneol improving LPS-induced brain injury following sepsis by regulating the HO-1, NOS2, and NF-κB/MAPK pathways (121). Well-known active compounds from Chinese herbal medicine, such as Pristimerin, Danshensu IIA, Cinnamyl alcohol, and Ginsenoside Rg3, have shown excellent anti-inflammatory effects. The information on other key individual active compounds from traditional Chinese medicine can be found in **Table 4**.

**Table 4:** Key monomeric components of traditional Chinese medicine with critical roles in sepsis.

Monomer Name	Main Source (Herb)	The role in sepsis	Main Effect	Key Pathways	Primary Targets/Proteins	References
Tanshinone IIA	*Salvia miltiorrhiza (Danshen)*	Inflammation & Neuroprotection	Protects neurons in sepsis, inhibits astrocyte and microglia activation.	NF-κB, MAPK	Inflammatory cytokines, glial activation markers	(122)
Cinnamyl Alcohol	*Cinnamomum cassia (Cinnamon)*	Inflammation	Inhibits NLRP3, reduces pro-inflammatory cytokines.	NLRP3, NF-κB, MAPK	NLRP3, IL-1β	(123)
Emodin	*Rheum palmatum (Rhubarb)*	Inflammation & Cardioprotection	Reduces LPS-induced myocardial injury by suppressing inflammation.	NLRP3 inflammasome, NF-κB	NLRP3, TNF-α, IL-1β	(124)
Glycyrrhizic Acid	*Glycyrrhiza uralensis (Licorice)*	Inflammation, Oxidative Stress & Apoptosis	Prevents septic AKI and ALI by reducing inflammation, oxidative stress, and apoptosis.	NF-κB, MAPK	TNF-α, IL-6, apoptotic proteins	(125)
Licorice Flavonoids	*Glycyrrhiza glabra/uralensis (Licorice)*	Inflammation	Inhibits HMGB1/TLR9 pathway, reducing inflammation	HMGB1/TLR9, NF-κB	HMGB1	(126)
Crocetin	*Crocus sativus (Saffron)*	Inflammation & Anti-apoptosis	Exhibits anti-inflammatory and anti-apoptotic effects; reduces cell death	MAPK, Bax/Bcl-2	Bax, Bcl-2	(127)
Ginsenoside Rg3	*Panax ginseng (Ginseng)*	Mitochondrial Protection & Inflammation	Mitigates sepsis-induced cellular and organ injury, enhances mitochondrial function.	AMPK	AMPK	(128)
Berberine	*Coptis chinensis (Huang Lian)*	Inflammation & Immunomodulation, Microbiota	Reduces inflammatory cytokine production and modulates gut microbiota	NF-κB, MAPK, AMPK	NF-κB, AMPK	(129)
Curcumin	*Curcuma longa (Turmeric)*	Inflammation & Oxidative Stress	Suppresses inflammatory cytokine production and oxidative stress	NF-κB, MAPK, Nrf2	NF-κB, COX-2, Nrf2	(129)
Resveratrol	*Polygonum cuspidatum/grapes*	Inflammation, Oxidative Stress & Immunity	Reduces systemic inflammation and protects mitochondria	SIRT1, NF-κB, AMPK	SIRT1, NF-κB	(130)
Baicalin	*Scutellaria baicalensis (Huang Qin)*	Inflammation & Oxidative Stress	Reduces inflammatory cytokine production, protects organ function	NF-κB, MAPK, PI3K/Akt	NF-κB, inflammatory cytokines	(131)
Andrographolide	*Andrographis paniculata (Chuanxinlian)*	Inflammation & Immunomodulation	Suppresses cytokine production, modulates immune response.	Nrf2/FSP1	Nrf2, FSP1	(132)
Oxymatrine	*Sophora flavescens (Ku Shen)*	Inflammation & Immunomodulation	Reduces cytokine production and immune cell activation	NF-κB, MAPK	NF-κB, IL-1β, TNF-α	(133)
Shikonin	*Lithospermum erythrorhizon (Zi Cao)*	Inflammation & Apoptosis Modulation	Reduces pro-inflammatory cytokines, triggers apoptosis in damaged cells.	NF-κB, MAPK, PI3K/Akt	NF-κB, Caspases	(134)

Traditional Chinese medicine compounds not only have the advantage of fewer side effects, but also interact directly with the gut microbiota after oral administration. With further research into the identification and pharmacological effects of these compounds, different key individual active ingredients with significant effects are becoming crucial strategies for the future treatment of sepsis.

## 7 Discussion

The core challenge in sepsis treatment lies in balancing immune mechanisms and inflammatory responses, particularly with the widespread use of antibiotics, which often disrupt the gut microbiota and further complicate and imbalance immune responses. This review systematically integrates the latest advancements in the regulation mechanisms of immunity, inflammation, and microbiota in sepsis treatment, emphasizing the pivotal role of the immune-inflammation-microbiota network in effective sepsis management. Unlike previous reviews that focus on individual mechanisms, we provide a holistic explanation of the dynamic balance between immune activation, inflammatory storms, and immune suppression triggered by infection. We present the dynamic process of functional phenotype transitions in immune cells (such as macrophages, neutrophils, and dendritic cells) and their impact on disease outcomes. Additionally, we explore the bidirectional effects of gut microbiota dysbiosis on systemic inflammation and immune dysfunction.

From a systemic perspective, the dynamic interplay between immune cell phenotype transitions, inflammatory storms, and immune suppression plays a key role in the progression of sepsis. Furthermore, the critical role of the microbiota and its metabolites, especially short-chain fatty acids, in immune regulation requires more attention. Although antibiotics are an important tool in the treatment of sepsis, their disruption of the gut microbiota can exacerbate endotoxin translocation and inflammatory responses, leading to multi-organ dysfunction. Unlike single-target therapies, traditional Chinese medicine enhances the multifaceted regulation of the immune system.

Traditional Chinese medicine offers the unique advantage of direct interaction with the gut microbiota through oral administration. Its components, such as flavonoids, polysaccharides, and saponins, not only exert significant anti-inflammatory, antioxidant, and immune-regulating effects but also promote the production of short-chain fatty acids (SCFAs), thereby restoring gut barrier function. Chinese patent medicines, herbal formulas, compound prescriptions, single herbs, and active ingredients (such as Pristimerin, tanshinone IIA, cinnamaldehyde, ginsenoside Rg3, etc.) play a role in regulating key signaling pathways like NF-κB, MAPK, JAK/STAT, PI3K/Akt, AMPK, and GSK-3β. These mechanisms present potential strategies to improve cure rates and patient prognosis. The multi-target combination therapy based on the immune-inflammation-microbiota network offers a new approach to addressing antibiotic resistance, uncontrolled inflammation, and immune suppression in sepsis treatment. It also opens new directions for the precision treatment of sepsis using traditional Chinese medicine. Personalized immunotherapy is a key focus in future sepsis research. Identifying critical targets through biomarker detection, tracking targeted modulation, and dynamically monitoring immune responses are essential for elucidating sepsis pathogenesis and guiding precise interventions.

Future research should further clarify the molecular mechanisms and key targets of the immune-inflammatory-microbial network regulatory system, aiming to improve the clinical outcomes of sepsis patients through multi-level interventions. In summary, this review integrates the latest research findings on immune, inflammatory, and microbial network regulation, not only enhancing the understanding of comprehensive treatment strategies for sepsis but also providing new theoretical foundations for future research and clinical applications.

## 8 Conclusion

Effective sepsis treatment relies on maintaining the balance between immunity, inflammation, and microbiota, as well as ensuring inter-organ coordination and homeostasis. Adopting a holistic network perspective of organ interactions and developing targeted therapies based on the immune-inflammation-microbiota network are critical strategies for enhancing treatment efficacy and improving patient outcomes.
